# The effect of an experiential learning program on motivations and activity involvement among dementia supporters in Japan

**DOI:** 10.1371/journal.pone.0244337

**Published:** 2020-12-28

**Authors:** Hiromi Arakawa, Tokie Anme

**Affiliations:** 1 Department of Nursing, International University of Health and Welfare, Ohtawara, Tochigi, Japan; 2 Faculty of Medicine, University of Tsukuba, Tsukuba, Ibaraki, Japan; Istituto Di Ricerche Farmacologiche Mario Negri, ITALY

## Abstract

**Purpose:**

The purpose of this study was to examine the effectiveness of an experiential learning program based on Kolb’s theory in increasing dementia supporters’ motivation and activity involvement within the community.

**Method:**

In this interventional study, the sample was divided into two groups. The intervention group underwent dementia supporter training and participated in an experiential learning program, which was conducted two weeks after the initial training session. The control group underwent only the dementia supporter training.

**Results:**

Kolb’s experiential learning model consists of four stages: concrete experience, reflective observation, conceptualization, and active experimentation. A total of 37 and 44 individuals constituted the intervention and control groups, respectively. The Wilcoxon signed-rank test revealed that there was a significant increase in motivation among the intervention group participants, when compared to the control group participants. Moreover, the activity rate was higher among the intervention group participants.

**Discussion:**

The experiential learning program was effective in increasing motivation levels and activity involvement among the dementia supporters.

**Conclusions:**

The experiential learning program for dementia supporters can be used to improve other volunteer and professional programs. Moreover, Kolb’s theory can be used to support individuals with dementia within the community.

## Introduction

In Japan, the older adult population is growing very rapidly. The percentage of individuals aged > 65 years was 28.4% in 2019. In other words, one out of four individuals were older than 65 years. The aging rate continues to grow, and one out of three individuals will be older than 65 years in 2036. In 2065, one out of 2.6 individuals will be older than 65 years [1]. Because of the aging of society, there is a larger number of older adults with dementia. Concurrently, the household structure in Japan has also changed. Those who live with an older adult constitute approximately half of all households in Japan. Households that consist of only one older adult couple comprise 32.3% of all households, and households in which older adults live alone constitute approximately 27.4% of all households [2].

Individuals with dementia tend to find it difficult to manage their money. Indeed, 40% of individuals with dementia and their families experience difficulties while shopping (e.g., buying too many products or wandering away from family members) [3]. Store employees and residents should acquire more knowledge about dementia symptoms and associated conditions to provide more effective assistance to community members with this condition. Local residents can also provide practical and emotional support to individuals with dementia [4–6].

In 2005, the Japanese government began recruiting and training dementia supporters (i.e., volunteers who can help individuals with dementia within the community). The dementia supporter system was the outcome of a Japanese policy, which aimed to manage the increasing prevalence of dementia [7]. To become a dementia supporter, one must attend a lecture to learn about the associated medical conditions, symptoms, and care protocols. By 2018, there were more than 10 million dementia supporters in Japan. Many reports [8, 9] have underscored the effectiveness of these lectures in helping dementia supporters understand dementia and increasing their motivation levels and activity involvement. However, the direct help rate remains low among dementia supporters; in other words, the supporters who were actively involved in the community accounted for only about 25% of all supporters [10, 11]. Dementia supporters are likely to continue to flourish alongside the growing population of older adults in Japan. Previous research reported that dementia supporters’ motivation and activity involvement are related, and those with higher motivation are much more likely to be involved in activities [10, 11].

On the other hand, experiential learning theory is reportedly effective in enhancing knowledge about dementia and mitigating negative attitudes toward individuals with dementia [12]. Specifically, they realize that individuals with dementia are not only care recipients but also caregivers, who have something to teach others [13].

This study examined the effectiveness of an experiential learning program in increasing dementia supporters’ motivation and activity involvement within the community. The program was designed based on Kolb’s experiential learning theory [14], which is based on Dewey’s learning theory [15].

Kolb’s experiential learning theory has been used for practitioners and students and not only for researchers, because the theory does not limit the target person for studied experiences, and the circular structure of the theory is easy to understand [16]. In educational training, college students created a bullying prevention program based on Kolb’s experiential learning theory and implemented it among high school students. The high school students’ evaluation items of positiveness, empathy, and cooperation increased, especially positiveness, which increased 30% after the program [17]. Additionally, one study reported that an educational program based on Kolb’s theory was more effective than one using a conventional program comparing outcomes of an observational cumulative rating scale: an educational scale evaluating teachers’ appropriate responses to their students’ remarks [18]. Additional research examines student teachers’ learning process in an educational practicum at an elementary school [19]. Based on the practicum records, students’ actual situation and teachers’ roles in elementary school were revealed as the learning process. From a student teacher’s records, child A monopolized a swing and never took turns with classmates in the schoolyard, so the student teacher scolded child A. Then, child A got angry and ran away from the student teacher (concrete experience). After that episode, the student teacher thought that she had put too much blame on child A (reflective observation), and she thought she needed supportive intervention considering child A’s feelings (abstract conceptualization). This abstract conceptualization led to her having a new experience with child A a few days later. The student teacher thought that, if she considered his previous behavior with attention to his feelings first, then she could teach him effectively. Through this experience, she increased her motivation in teaching.

The program aimed to enhance participants’ knowledge and help them actively progress through the four stages outlined by Kolb: concrete experience, reflective observation, abstract conceptualization, and active experimentation [20].

## Objectives and hypothesis

### Objectives

The objective of this study was to examine the effectiveness of the experiential learning program (based on Kolb’s theory) in increasing dementia supporters’ motivation and activity involvement within the community.

### Hypothesis

It was hypothesized that the intervention group (i.e., those who participate in the experiential learning program) will be more motivated and active within the community than the control group (i.e., those who do not participate in the experiential learning program).

## Methods

### Research design

In this interventional study, the participants were divided into two groups.

1. The intervention group participated in a dementia supporter training session and the experiential learning program, which was implemented two weeks after the initial training session.
2. The control group participated in a dementia supporter training session but not the experiential learning program.

The participants were assessed at four time points: before the dementia supporter training session, after the session, two weeks after the session, and three months after the session.

### Participants

The researcher received information about the dates and venues of the dementia supporter training sessions from the session organizer, and she attended the sessions and recruited participants. The inclusion criterion was completion of the training session. Those with a diagnosis of dementia and those who were unable to communicate with another person were excluded.

The participants were assigned to the intervention and control groups by assigning even (intervention group) and odd (control group) numbers to all the training sessions conducted in O City between February and June 2019.

### Operational definitions

#### Dementia supporter

Individuals who participated in the dementia supporter training session offered by their prefectures, municipalities, occupational organizations, or businesses were considered to be dementia supporters. The two-hour training session equipped them with information about associated medical conditions, symptoms, and care protocols for individuals with dementia.

#### Dementia supporter activities

Care for individuals with dementia. The supporters provided assistance with daily life activities (e.g., helping them take out the trash, helping them find their way home, and engage in empathic communication to individuals with dementia.

### Program

#### Intervention group

**Fig 1:**
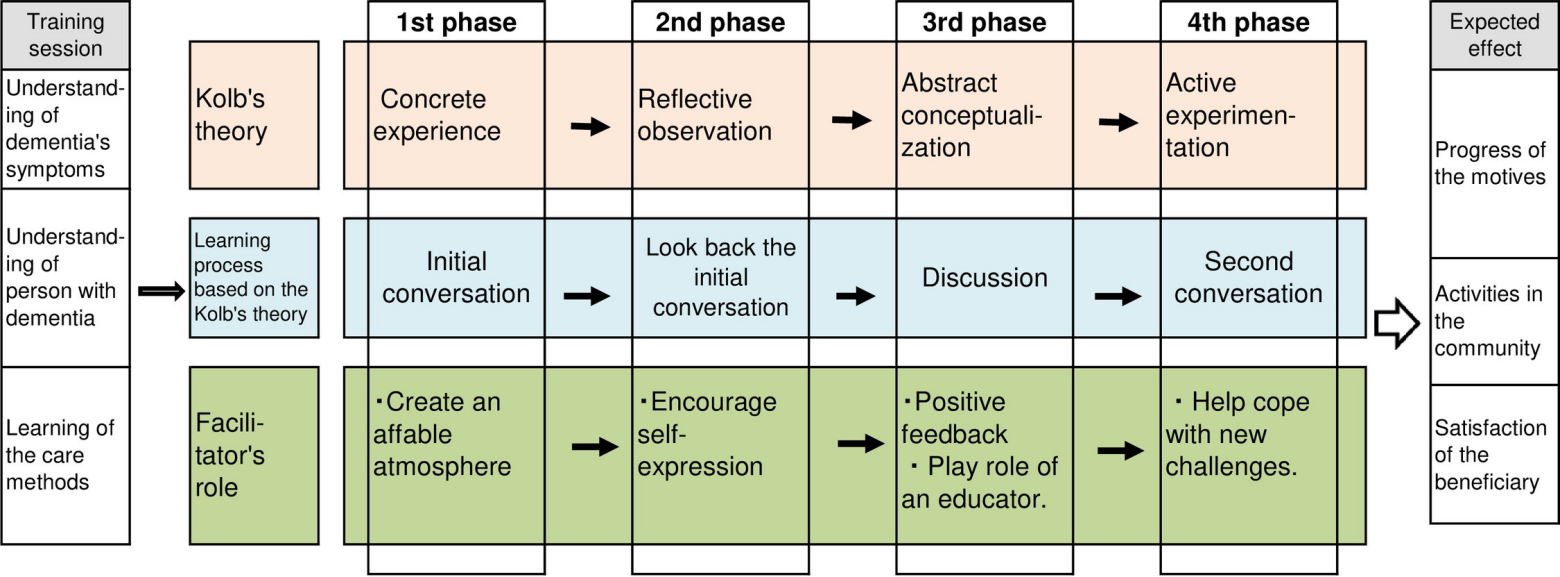
Experiential learning methods based on Kolb's theory.

The experiential learning program was designed based on Kolb’s model (Fig 1), which consists of four stages. The first phase focused on concrete experience (e.g., initiating a conversation with an individual with dementia). The second phase focused on reflective observation. Specifically, the dementia supporters reflected upon their prior interactions with individuals with dementia. The third phase focused on abstract conceptualization, which was facilitated through discussion. The fourth phase focused on active experimentation. Accordingly, the supporters shared a second conversation with individuals with dementia. This experiential learning program has been used in several fields, including business, and found to be effective [21, 22].

#### Control group

Following the training session, the control group received neither another intervention nor homework assignments. They went about their daily lives as usual.

#### Intervention content

This study was conducted in a daycare center. The program began at 9 am and lasted for approximately three to three-and-a-half hours. Each dementia supporter talked with a person with dementia. There were no prompts regarding conversational topics. As noted earlier, there were two interactions (i.e., initial and second conversation), each of which lasted for approximately 60 minutes. The supporters were allowed to only converse with individuals with dementia. To prevent untoward incidents, they did not provide physical care.

*Preparation*. An orientation to the schedule of the day and important notices were provided to the supporters. A daycare center manager played the role of a facilitator in this experiential learning program.

*The first phase*. The dementia supporters freely interacted with individuals with dementia. Prior to the interactions, they were asked to role-play three roles based on a case study: a person with dementia, a dementia supporter, and an observer. This activity was conducted to help them start the conversation with ease.

After the role-play activity, the supporters were introduced to an individual with dementia. During this phase, the facilitator tried to create an affable atmosphere.

*The second phase*. Once the initial conversations had come to an end, the supporters were invited to assemble in a meeting room and reflect upon their conversations. They were asked to focus on the words that had been spoken to the individual with dementia to enhance their empathy for individuals with dementia. During this phase, the facilitator encouraged self-expression.

*The third phase*. Discussions with the facilitator helped the participants gain a broader and deeper understanding of their experiences and apply what they had learned in their subsequent conversation. For example, when a supporter reported that the individual with dementia had repeatedly shared the same story, the facilitator provided positive feedback to the supporter (i.e., for listening to the individual with dementia without expressing annoyance). Further, the facilitator suggested that he or she can ask the individual with dementia to talk about a new topic to expand the scope of their conversation and elicit a new story from him or her. During this phase, the facilitator played the role of an educator.

*The fourth phase*. This phase aimed to help the supporters utilize what they had learned during the third phase. During this phase, the facilitator tried to help the supporters face and cope with new challenges.

### Measurements

#### Demographic information

The following demographic characteristics were assessed: sex, age, occupation, community participation, prior experience in communicating with individuals with dementia, and self-rated health.

#### Motivation for supporting individuals with dementia

Motivation was assessed using two items that corresponded to the Visual Analog Scale (VAS) and Sense of Coherence (SOC) Scale [23]. These two items were extracted based on concept analysis of *dementia supporters* [10, 24, 25]. The SOC Scale was developed by Antonovsky (1987) and was conceptualized as health generation, known as the ability to cope with stress and maintain good health. In the previous research, an intergenerational exchange program was implemented in which an elderly person read picture books for children, and, after the program, the elderly person’s SOC score increased. It was thought that the elderly people felt a sense of worth through their experience [26]. Further, past studies have found that dementia supporters’ activities are influenced by their motivation levels [10]. The VAS ranges from 0 (low motivation) to 10 (high motivation). The short form of the Japanese version of the SOC Scale (SOC-13J) was used in this study. The SOC-13J consists of three subscales: comprehensibility, manageability, and meaningfulness. This scale consists of 13 questions, each of which can receive scores that range from 0 to 7. Total scores can range from 0 to 91. Higher scores indicate stronger motivation to engage in a particular activity.

#### Knowledge

Knowledge was assessed using ten questions, which pertained to associated medical conditions, symptoms, and dementia care. One score was awarded for each correct answer. The maximum possible score was 10.

#### Statistical analysis

Participant motivation to support individuals with dementia (i.e., VAS and SOC-13J) and knowledge were assessed. The Wilcoxon signed-rank test, Mann-Whitney U test, and χ^2^ test were used to examine differences before and after the dementia supporter training session, two weeks later, and three months after the session (in each group). All analyses were conducted using SPSS version 23.

#### Interview

After the experiential program, supporters were interviewed. The interview consisted of questions regarding the thoughts and learning related to communicating with people with dementia. The data from the interview were analyzed using content analysis.

## Ethical issues

This study was approved by the ethics committee of the University of Tsukuba (No. 1241). All the participants provided written informed consent prior to participation. During the experiential learning program, supporters were introduced to an individual with dementia who had consented (or whose family had consented) to participate in this program.

## Results

In total, 37 individuals were in the intervention group, and 44 were in the control group. Those who did not participate in the follow-up evaluation were excluded.

Participant demographic characteristics are shown in Table 1. In each group, more than 80% of the participants were women. In both the groups, the unemployment rate was approximately 30%. There were no significant demographic differences between the intervention and control groups.

**Table 1:** Demographic information.

		Intervention		Control		p
		n	%	n	%	
Participants	male	6	16.2	10	22.7	0.46^a)^
	female	31	83.8	34	77.3	
Age		(Avg:67.6, SD: 10.7)		(Avg: 63.1, SD: 15.2)		0.23
Occupation	Unemployed	10	27.0	13	29.5	0.11
	housewife	19	51.4	10	22.7	
	part-time job	3	8.1	4	9.1	
	employee	4	10.8	11	25.0	
	self employed	1	2.7	5	11.4	
	Other	0	0.0	1	2.3	
Participation for neighborhood association	not at all	4	10.8	8	18.2	0.27
	seldom	7	18.9	9	20.5	
	sometimes	8	21.6	8	18.2	
	almost	5	13.5	9	20.5	
	always	13	35.1	10	22.7	
Participation for volunteers	not at all	4	10.8	10	22.7	0.09
	seldom	9	24.3	14	31.8	
	sometimes	11	29.7	7	15.9	
	almost	4	10.8	7	15.9	
	always	9	24.3	6	13.6	
Neighborhood relationship	not at all	2	5.4	2	4.5	0.69
	seldom	5	13.5	11	25.0	
	sometimes	11	29.7	11	25.0	
	almost	10	27.0	7	15.9	
	always	9	24.3	13	29.5	
Experiences with the dementia	not at all	4	10.8	7	15.9	0.78
	seldom	3	8.1	6	13.6	
	sometimes	10	27.0	8	18.2	
	almost	8	21.6	7	15.9	
	always	12	32.4	16	36.4	
Activities	not-active	21	56.8	29	65.9	0.40^a)^
	active	16	43.2	15	34.1	
Self-rated health	excellent	7	18.9	8	18.2	0.71
	well	28	75.7	32	72.7	
	poor	2	5.4	4	9.1	

Mann-Whitney U test

^a)^ = χ2 test.

The Wilcoxon signed-rank test (Table 2) revealed that, among the intervention group participants, there was a significant increase in motivation after the training session, after two weeks, and after three months. Moreover, among the control group participants, there was a significant increase in motivation after the session but not after two weeks and after three months. With regard to the SOC-13J scores, among the intervention group participants, there was a significant increase in the meaningfulness subscale scores after two weeks and after three months. Among the control group participants, there was no significant increase in SOC-13J scores between any of the time points.

**Table 2:** Changes in motivation levels among dementia supporters.

		Before		After		p	2 weeks		p	3 months		p
		Mdn	25–75%	Mdn	25–75%		Mdn	25–75%		Mdn	25–75%	
**motieves**	Intervention	6.40	4.85–7.80	7.10	5.50–8.20	<0.001	7.50	5.30–8.75	0.005	7.00	5.30–8.85	0.046
** **	Control	5.60	4.45–7.13	6.65	4.80–7.68	0.004	5.70	4.50–7.08	0.343	5.10	3.55–6.70	0.184
**SOC:Co**	Intervention	24.0	22.0–27.5	24.0	21.5–27.5	0.789	25.0	22.5–29.0	0.254	25.0	21.5–29.0	0.792
**Ma**		19.0	17.0–22.0	18.0	16.0–22.0	0.234	19.0	16.0–22.0	0.929	19.0	15.5–20.5	0.255
**Me**		21.0	18.5–23.0	21.0	17.5–23.5	0.775	22.0	19.0–24.5	0.038	23.0	19.0–25.0	0.042
**SOC:Co**	Control	22.0	20.0–24.0	22.5	19.0–25.0	0.861	23.0	20.0–25.0	0.435	23.0	19.0–26.0	0.663
**Ma**		17.0	15.0–20.0	17.0	15.3–20.0	0.664	17.0	15.0–19.0	0.296	17.5	15.0–19.8	0.743
**Me**		20.0	16.0–22.8	18.0	16.3–21.8	0.147	19.5	17.0–21.8	0.945	19.0	17.0–21.8	0.573

Wilcoxson singed ranks test

SOC: comprehensibility; Co, manageability; Ms, meaningfulness; Me

Mdn: Median.

The Wilcoxon signed-rank test (Table 3) also showed that the knowledge levels of the intervention group had increased after the session, after two weeks, and after three months. Among the control group participants, knowledge levels had increased after the session and after two weeks but not after three months.

**Table 3:** Changes in knowledge among dementia supporters.

	Before				After				p	2 weeks				p	3 months				p
	Avg	SD	Mdn	25–75%	Avg	SD	Mdn	25–75%		Avg	SD	Mdn	25–75%		Avg	SD	Mdn	25–75%	
Intervention	8.5	1.0	9.0	8.0–9.0	9.2	0.9	9.0	9.0–10.0	0.001	9.2	0.9	9.0	9.0–10.0	0.001	8.9	0.9	9.0	8.0–10.0	0.022
Control	8.3	1.3	9.0	8.0–9.0	8.7	1.1	9.0	8.0–9.0	0.018	8.7	1.2	9.0	8.0–9.0	0.031	8.6	1.0	9.0	8.0–9.0	0.106

Wilcoxson singed ranks test

Avg: Average, SD: Standard deviation, Mdn: Median.

With regard to activity involvement (Table 4), 43.2% of the intervention group participants were active before the program, and this figure increased to 70.3% after the program. There was no change in the percentage of control group participants who were active before and after the program (34.1%). There was a significant difference in participant activity rates between the intervention and control groups.

**Table 4:** Changes in activity involvement rates among dementia supporters.

	Before					3 months				
	Intervention		Control		p	Intervention		Control		p
	Participant	%	Participant	%		Participant	%	Participant	%	
Not active	21	56.8	29	65.9	0.399	11	29.7	29	65.9	0.001
Active	16	43.2	15	34.1		26	70.3	15	34.1	
Total	37	100	44	100		37	100	44	100	

χ2 test.

Interview results are shown in Table 5: Dementia supporter's thoughts and learning through the experiential learning program. [] indicates the category, and < > indicates the sub-category. Six categories were extracted: [concern before the program], [proper understanding of people with dementia], [respect for the person with dementia’s motivation to communicate], [difficulty of communication], [possible support], and [application for community support]. Additionally, 18 sub-categories were extracted.

**Table 5:** Dementia supporters' thoughts and learning through the experiential learning program.

Category	Sub-category
concern before the program	concern about the person with dementia’s stress from supporters
proper understanding of people with dementia	understanding of short- and long-term memory
	understanding of daily living for the person with dementia
	change from a negative image to a positive one
	pleasant and interesting experience
respect for the person with dementia’s motivation to communicate	support the person with dementia to talk
	extraction of positive feelings
	constantly keep an eye on responses from the person with dementia
	provide a topic for conversation
difficulty of communication	reconsideration of conversation with the person with dementia on only the supporter's topic of interest
	difficulty of communicating with an uncommunicative person
	difficulty of conversation with multi-person talk about the dementia.
possible support	awareness in everyday life in the community
	preserve enough time for support the person with dementia
	support for the family of the person with dementia
	cooperate with other dementia supporters
application for community support	content that connects to activity in the community
	advantage of experiential learning

## Discussion

The experiential learning program conducted in this study was effective in increasing the motivation levels of dementia supporters. Among the intervention group participants, there was an increase in motivation. Further, at three-month follow up, their motivation levels were higher than those of the control group participants, who demonstrated improvements only immediately after the training session.

There are four possible explanations for why an increase in motivation was observed. First, the supporters had enough time to prepare for and understand the program and prepare to communicate with individuals with dementia through engagement in role-play activities. Yamada [27] found that readiness plays an important role in maximizing the effects of education on learners. Moreover, it is important for facilitators to understand their participants.

Second, the dementia supporters possessed a basic understanding of dementia and were aware of the importance of caring for community members with dementia because of prior training. Kage [28] found that, when individuals realize the importance and meaningfulness of their actions, their motivation levels tend to remain high. Therefore, implementing the experiential learning program after the training session may have enhanced its effectiveness. Additionally, during the second phase (i.e., reflection), the supporters could connect their experience of interacting with individuals with dementia to what they had learned during the training session. It is necessary for learners to connect their experiences to what they have learned in the past [29].

According to the interview results, [proper understanding of people with dementia], <understanding of short- and long-term memory>, and <understanding of daily living of the person with dementia> were extracted, and these categories indicated that the supporters were aware that the person with dementia likely talked of their old days repeatedly. This experiential learning program connected academic knowledge and learning in the field.

Third, the dementia supporters presumably found the experience of communicating with individuals with dementia to be pleasant and interesting; this may have increased their motivation levels. Kage [28] found that, when individuals have a good time and have fun, their motivation levels increase. Moreover, dementia supporters received positive feedback from their facilitator; this may have helped them stay positive and motivated.

In the interview, shown in [proper understanding of people with dementia] and <pleasant and interesting experience>, there were dementia supporters who had not talked with a person with dementia before the program. They considered the program as a precious experience, and they had a good time talking with person with dementia. Some supporters said the program made them remember their parent who had been diagnosed with dementia and died. The supporters said they spent a peaceful time talking with the person with dementia.

Fourth, there was a change in the beliefs of the dementia supporters. Before the experiential learning program, the dementia supporters believed that there were limitations to what individuals with dementia can achieve (e.g., during conversations), and they held negative attitudes toward individuals with dementia. During the intervention, especially the second phase, the supporters realized that individuals with dementia can be outgoing, communicate with others with ease, and independently engage in many activities. Moreover, during the third phase, discussions with the facilitator helped the supporters gain a better understanding of their experiences. Consequently, they realized that individuals with dementia should not be stereotyped.

According to the interview results, [proper understanding of people with dementia] and <change from a negative image to a positive one> also support the effect of this program.

The preprogram SOC-13J scores of the participants (intervention group: 64.7, control group: 58.3) were slightly higher than those of the average adult (54.0–57.0) [30]. Life experiences influence one’s SOC. Thus, participant age (63–67 years) may explain the high SOC-13J scores that were observed in this study. After the program, the intervention group obtained higher meaningfulness scores but not comprehensibility and manageability scores. Comprehensibility refers to the perception of situations as orderly and predictable. Manageability refers to the belief that one can independently manage different situations. Meaningfulness refers to the experience of meaning, even in difficult situations [23]. Past studies have found that self-promotion program and intergenerational exchanges enhance one’s SOC [24, 29]. In this study, the experiential learning program addressed prejudices and preconceived notions; therefore, the dementia supporters’ SOC may have improved. The experiential program fostered a sense of existential meaningfulness in the participants. The symptoms of dementia vary from person to person [31], and, in an aging society, everyone is vulnerable to the eventual onset of dementia. This program increased the motivation levels of dementia supporters by helping them find greater meaning in life.

## Limitations

This study was conducted in only one daycare center, and only one facilitator managed this program. Future studies should implement this program across several daycare centers and recruit many facilitators to test the robustness of the present findings.

## Conclusion

This program aimed to raise dementia supporters’ motivation and their activity involvement in the community and to examine the effectiveness of the experiential learning program. People in the intervention group increased their motivation and activity involvement compared to the people in the control group. The experiential learning program had significant positive effects on dementia supporters.

Kolb’s experiential learning model consists of four stages: concrete experience, reflective observation, conceptualization, and active experimentation. This study was the first practical application of Kolb’s theory to training for dementia supporters. The findings indicated that Kolb’s theory is useful for supporting individuals with dementia and can be used to improve other volunteer and professional programs.

## Supporting information

S1 Data (XLSX)
